# Inflammation associated with monocyte/macrophage activation and recruitment corresponds with lethal outcome in a mouse model of Crimean-Congo haemorrhagic fever^1^

**DOI:** 10.1080/22221751.2024.2427782

**Published:** 2024-11-08

**Authors:** Teresa E. Sorvillo, Jana M. Ritter, Stephen R. Welch, JoAnn D. Coleman-McCray, Katherine A. Davies, Heather M. Hayes, Scott D. Pegan, Joel M. Montgomery, Éric Bergeron, Christina F. Spiropoulou, Jessica R. Spengler

**Affiliations:** a Viral Special Pathogens Branch, Division of High Consequence Pathogens and Pathology, Centers for Disease Control and Prevention, Atlanta, GA, USA; b Infectious Disease Department, CDC Foundation, Atlanta, GA, USA; c Infectious Diseases Pathology Branch, Division of High Consequence Pathogens and Pathology, Centers for Disease Control and Prevention, Atlanta, GA, USA; d United States Department of Agriculture, Agricultural Research Service, Zoonotic and Emerging Disease Research Unit, National Bio and Agro-Defense Facility, Manhattan, KS, USA; e Division of Biomedical Sciences, University of California Riverside, Riverside, CA, USA

**Keywords:** Crimean-Congo haemorrhagic fever virus, CCHF, Mice, Inflammation, Tropism, Macrophage activation

## Abstract

Crimean-Congo haemorrhagic fever virus (CCHFV) causes human disease ranging from subclinical to a fatal haemorrhagic syndrome. Determinants of CCHF pathogenesis are largely unknown and animal models that recapitulate human disease are limited. A recently described mouse model uses a monoclonal antibody (mAb 5A3) targeting the interferon (IFN) alpha/beta receptor to suppress type I IFN responses, making animals transiently susceptible to infection. To advance utility of this model, we investigated effects of challenge route, timing of 5A3 delivery, mouse sex and age, and virus strain on clinical course and outcome. C57BL/6J mice received mAb 5A3 −1, 0, or −1/+1 days post-infection (dpi). Subsets were challenged with CCHFV strain Turkey04 or IbAr10200 subcutaneously or intraperitoneally, and serially euthanized 3- and 7-dpi, when meeting euthanasia criteria or at study completion (14 dpi). CCHFV-IbAr10200-infected mice almost uniformly succumbed to infection, whereas CCHFV-Turkey04-infected mice transiently lost weight but survived. These results were consistent regardless of mAb timing or route of challenge. Viral replication and dissemination were comparable between the two strains at 3 dpi. However, in the plasma and livers of non-survivors, expression of proinflammatory cytokines/chemokines that correspond with macrophage activation and recruitment were significantly elevated. Lethal disease was also associated with elevated levels of macrophage activation marker CD163 in plasma. Further, mouse macrophages were more permissive to IbAr1200 infection *in vitro*, suggesting tropism for these cells may influence pathogenesis. Our data suggest that early inflammation may be a critical determinant of CCHF outcome and therapeutics to control inflammation may be worthwhile targets for future investigation.

## Introduction

Crimean-Congo haemorrhagic fever virus (CCHFV) is a pathogen of public health importance that causes a wide spectrum of human disease including fatal haemorrhagic illness. This tickborne *Orthonairovirus* (family *Nairoviridae*) has a wide endemic range across numerous countries in Europe, Asia, and Africa, and is considered a WHO priority pathogen due to its increasing incidence and lack of FDA-approved vaccines or therapeutics [1]. Human case fatality rates of Crimean-Congo haemorrhagic fever (CCHF) vary widely, from 4 to 40%, although mild cases are likely underreported and thus underrepresented in these numbers [2,3]. Clinical manifestations of CCHF often include fever, headache, myalgia, and photophobia. These can be accompanied by sore throat, nausea, vomiting and diarrhoea, and, occasionally, conjunctivitis and jaundice [4]. Haemorrhagic manifestations are present in only a subset of cases and can range from a mild petechial rash to widespread ecchymoses [5,6].

The cause of differing clinical symptoms and outcomes is unknown but may be due to the high genetic diversity of the virus which is categorized into 6 distinct clades [7]. Studies into the host’s contribution to CCHF pathogenesis suggest that fatal human disease correlates with elevated levels of systemic inflammation, specifically the analytes MCP-1 (CCL-2), CXCL10 (IP-10), IL-6, and TNF-alpha [8–11]. In fact, in a lethal mouse model of CCHF, treatment with a monoclonal antibody (mAb) to reduce TNF-alpha was partially (50%) protective [12]. Despite these data, significant gaps in our understanding of CCHF pathogenesis remain.

Animal models to recapitulate the spectrum of human disease are critical tools for addressing these gaps but have been historically limited for CCHF. Mice serve as the only small animal and uniformly lethal models of CCHF but require either virus adaptation or disruption of type I interferon (IFN-I) signalling (*e.g.* IFNAR^-/-^, STAT-1^-/-^) [13–15]. A new mouse model utilizes a monoclonal antibody (mAb 5A3) targeting the IFNAR-1 subunit of the IFN-alpha/beta receptor [16] to transiently suppress IFN-I responses, making immunocompetent animals susceptible to lethal CCHFV infection [17]. The model, referred to as the immunosuppression (IS) model, maintains some of the same limitations as IFNAR^-/-^ mice where a broad range of innate and adaptive immune signalling pathways may be altered by the lack of IFN-I responses [18]. However, this model is an important advancement for the field as it allows the use of knockout mouse strains to conduct mechanistic studies on viral pathogenesis or immune correlates of protection.

Here, we advance utility of the IS model by investigating the effect of parameters not previously evaluated on CCHFV pathogenesis and disease outcome. These include timing of mAb 5A3 delivery, CCHFV challenge route, mouse sex and age, and CCHFV strain. Further, through investigations of both lethal and non-lethal disease models, we demonstrate utility of the IS model in identifying biomarkers associated with improved clinical outcomes after CCHFV infection.

## Materials and methods

### Biosafety and ethics statement

Experiments involving CCHFV were conducted in the BSL-4 laboratory at the Centers for Disease Control and Prevention (CDC; Atlanta, GA, USA). Experiments involving cDNA-encoding viral sequences were performed in accordance with approved Institutional Biosafety Committee protocols. Animal studies were conducted in compliance with the *Guide for the Care and Use of Laboratory Animals* and approved by the CDC Institutional Animal Care and Use Committee (IACUC #3102, 3342, 3343). The CDC is fully accredited by AAALAC International.

### Viruses and cells

Recombinant CCHFV IbAr10200 (recIbAr10200; Africa-3 clade) is based on the sequence of a Nigerian tick isolate passaged 9× in suckling mouse brain and 3× in HepG2 cells (GenBank: KJ648914, KJ648915, and KJ648913). Recombinant IbAr10200 was rescued in Huh7 cells and passaged 3× in BSR-T7/5 cells (Bergeron et al., 2015). CCHFV Turkey-200406546 (Turkey04; Europe-1 clade) was isolated from a hospitalized patient with unknown outcome, passaged 1× in suckling mouse brain, and 1× in SW-13 cells (GenBank: KY362517, KY362519, KY362515)). BSRT7/5 cells (baby hamster kidney cells stably expressing T7 polymerase) were kindly provided by K.K. Conzelmann, Ludwig-Maximilians-Universität, Munich, Germany. HuH-7 cells human hepatoma cell line were obtained from APATH LLC. PMJ2-R (peritoneal macrophage cell line derived from C57BL/6J mice) and THP-1 cells (human monocyte cell line) were obtained from ATCC. All virus and cell line stocks were confirmed to be free of mycoplasma using MycoAlert PLUS detection kit (Lonza LT07) and next-generation sequencing.

### Antibodies

Anti-mouse IFNAR-1 purified *in vivo* GOLD functional grade monoclonal antibody (5A3) was purchased from Leinco Technologies, Inc (I-401, Lot #s 0622L675, 0521L200, 0822L285, 0721L710, 1221L440, 0323L565). InVivoMAb mouse IgG1 (isotype control) antibody was purchased from Bio X Cell, Inc (BE0083, Lot # 785121M1). Anti-Folate receptor beta IgG2a (CL10) antibody was purchased from Absolute Antibody (AB00498-2.0-BT). Diluent for all antibodies administered *in vivo* was sterile PBS alone.

### Mice

C57BL/6J mice (The Jackson Laboratory, 000664; ages ranging from 6 to 83 weeks; mixed males and females) were housed in a climate-controlled laboratory with a 12 h day/night cycle; provided sterile rodent chow (LabDiet, 5010) and water *ad libitum*; and group-housed on autoclaved corn cob bedding (Anderson Lab Bedding, Bed-o’Cobs ¼″) with cotton nestlets in an isolator-caging system (Tecniplast, GM500 cages) with a HEPA-filtered inlet and exhaust air supply. Mice were evaluated daily for clinical signs of disease and assigned a score ranging 0–10 based on the following criteria: piloerection, hunched posture, hypoactivity, percent weight loss, abnormal respiration, dehydration, and neurological signs (ataxia, paresis). Euthanasia criteria were met when weight loss exceeded 25% from baseline at 0 dpi and/or the clinical score reached 10. Mice were humanely euthanized via isoflurane exposure followed by cervical dislocation at the serial timepoints indicated or when meeting euthanasia criteria according to protocols approved by CDC’s IACUC.

### RT-qPCR

RNA was extracted from EDTA whole blood (50 µL), oral and rectal mucosal swabs, and homogenized tissue (liver, spleen, gonad [testis/ovary], kidney, heart, lung, eye, brain) in MagMAX lysis buffer. MagMAX Pathogen RNA/DNA kits (Thermo Fisher Scientific, 4462359) were used in conjunction with the 96-well ABI MagMAX extraction platform; RNA was eluted into 75 µL of elution buffer. Viral RNA was quantified using a primer/probe set targeting either the NP open reading frame of the S genomic segment of CCHFV strain IbAr10200 (forward 5′-CAG GAC ATG GAC ATA GTG GC-3′; reverse: 5′-ATT GCC CTT GAC GTT GTA GG-3′; probe: 5′-CCC TTG TTG GCA AGC AAT CCC-3′) or Turkey04 (forward: 5′-CAA CAG GCT GCT CTC AAG TG -3′; reverse: 5′-CAA TTT CGC CAG GGA CTT TA-3′; probe: 5′-ACA CGG CAG CCT TAA GCA ACA A-3′ [all IDT]) using the SuperScript III Platinum One-Step RT-qPCR kit (Thermo Fisher Scientific, 11732088). The average Ct value of a housekeeping gene (18S [pilot study] or Gusb and Ppia [subsequent studies]) was calculated for each tissue type and used to normalize the Ct values from each sample. vRNA copy numbers were quantified via standard curve generated from an RNA standard of known concentration (IDT). Data are reported as S genome copy number/µL RNA.

Gene expression was quantified using a custom RT^2^ Profiler PCR Array (Qiagen, 330171). cDNA synthesis was performed using 750 ng (liver) or 1 µg (spleen) total RNA per tissue and the RT^2^ First Strand Kit (Qiagen, 330404); qPCR was performed using RT^2^ SYBR Green Mastermix (Qiagen, 330503). Data were analysed using GeneGlobe Data Analysis Center software (Qiagen). Genomic DNA contamination threshold was Ct ≥ 35. Ct values from each sample were normalized to levels of 2 housekeeping genes: actin beta (Actb) and beta-2 microglobulin (B2 m). Gene expression data are reported as fold change compared to tissue from age-matched uninfected C57BL/6J control animals.

### Virus isolation and quantification

Oropharyngeal and rectal swabs (Puritan Medical Products, 25–800 1PD 50) collected for virus isolation were placed in serum-free DMEM supplemented with 2× antimycotic/antibiotic (Gibco, 15240062) and allowed to sit at room temperature (RT) for 15–20 min prior to swab removal and storage at −80°C. For virus quantification, samples were centrifuged and plated (100 µL/well) in triplicate onto BSR-T7/5 cells seeded into 12-well plates. Plates were incubated at 37°C and rocked every 15 min for 1 h. DMEM (1 mL) supplemented with 5% FBS and 2× antibiotic/antimycotic was added to each well before plates were incubated at 37°C for 5 days. Plates were fixed with 4% formaldehyde and permeabilized with 0.1% Triton-X-100, followed by immunostaining with a polyclonal rabbit anti-NP CCHF antibody (1:2500; IBT, 04-0011) and goat anti-rabbit secondary antibody (Alexa Fluor 488; Invitrogen, A-11008). Samples were determined to be positive for CCHFV if one or more fluorescent foci were visually present. Positive samples were further analysed via immunofluorescent TCID_50_ assay to quantify infectious virus using the Reed–Muench method.

### Histology and immunohistochemistry

Tissue specimens were fixed in 10% neutral buffered formalin. Tissues were processed for paraffin embedding, sectioning, and staining with haematoxylin and eosin. Immunohistochemical assays were performed using indirect immunoalkaline phosphatase detection; 4 μm tissue sections were placed on slides, deparaffinized in xylene, and rehydrated through graded alcohol solutions. Colorimetric detection was performed using the Mach 4 AP Polymer kit (Biocare Medical, M4U536). Staining procedures were performed at RT. Slides were digested with 0.1 mg/mL proteinase K in 0.6M tris/0.1% CaCl_2_ for 15 min, then blocked in Background Punisher (Biocare Medical, BP974) for 10 min and incubated with a rabbit anti-CCHFV N pAb (IBT, 04-0011) diluted 1:1000 for 30 min. Mach 4 AP polymer was applied for 30 min. The antibody/polymer conjugate was visualized by applying Sigmafast Fast Red Chromogen (Millipore Sigma) to tissue sections for 30 min. Slides were counterstained in Mayer’s haematoxylin (PolyScientific) and stained blue with lithium carbonate (Polysciences Inc). Slides were coverslipped using aqueous mounting medium (Polysciences Inc).  The following representative tissue sections were evaluated: one longitudinal section for brain (midsaggital), heart, eye, kidney, adrenal gland, liver (median lobe, including gallbladder), spleen, ovary, oviduct, uterus (one horn), urinary bladder, testis, epididymis, stomach (including glandular and nonglandular portions), jejunum, ileum, caecum, colon; one horizontal section for pancreas (right lobe), male accessory sex glands en bloc; one section of lungs en bloc (embedded ventral surface down).

### Clinical chemistry

Whole blood from each animal was collected peri-mortem via intracardiac bleed, placed in lithium heparin, and immediately analysed via the Comprehensive Metabolic panel on the Piccolo Xpress analyzer (Abaxis).

### Cytokine/chemokine analyses

EDTA plasma samples from each animal were gamma-irradiated (5.0 × 10^6^ rad dose) and analysed (25 µL) using the ProcartaPlex Mouse Th1/Th2 Cytokine and Chemokine 20-plex panel (Thermo Fisher Scientific, EPX200-26090-901). Data were read using the Luminex 200 analyzer. All analytes are calculated based on standard curves and reported as absolute values (pg/mL).

### CD163 ELISA

Gamma-irradiated (5.0 × 10^6^ rad dose) EDTA plasma samples were analysed (1:100) using a Mouse CD163 SimpleStep ELISA kit (Abcam, ab272204). Optical density (OD) values were read at 450 nm on the Synergy Neo2 (BioTek) microplate reader. Final analyte concentrations were extrapolated from a standard curve. Plasma samples (*n* = 4) from age-matched naive C57BL/6J mice were analysed in parallel to determine baseline levels of CD163 in the plasma of healthy mice.

### In vitro experiments

THP-1 cells were differentiated by incubating with 5 ng/mL phorbol 12-myristate 13-acetate (PMA; Tocris Bioscience, 1201) for 24 h, followed by incubation with standard RPMI medium for 72 h, per Baxter *et al.* 2020 [19]. When indicated, THP-1 and PMJ2-R cells were polarized (classical, M1 phenotype) by incubating with 250 ng/mL lipopolysaccharide and 20 ng/mL mouse or human IFN-gamma (R&D Systems, 485-MI-100/CF or 285-IF-100/CF) for 48 h [19,20].

### Statistical analyses

All statistical analyses were conducted using GraphPad Prism version 9.1.2.

## Results

### Lethal outcome in transiently immunosuppressed mice is dependent on CCHFV strain

**Figure 1.**
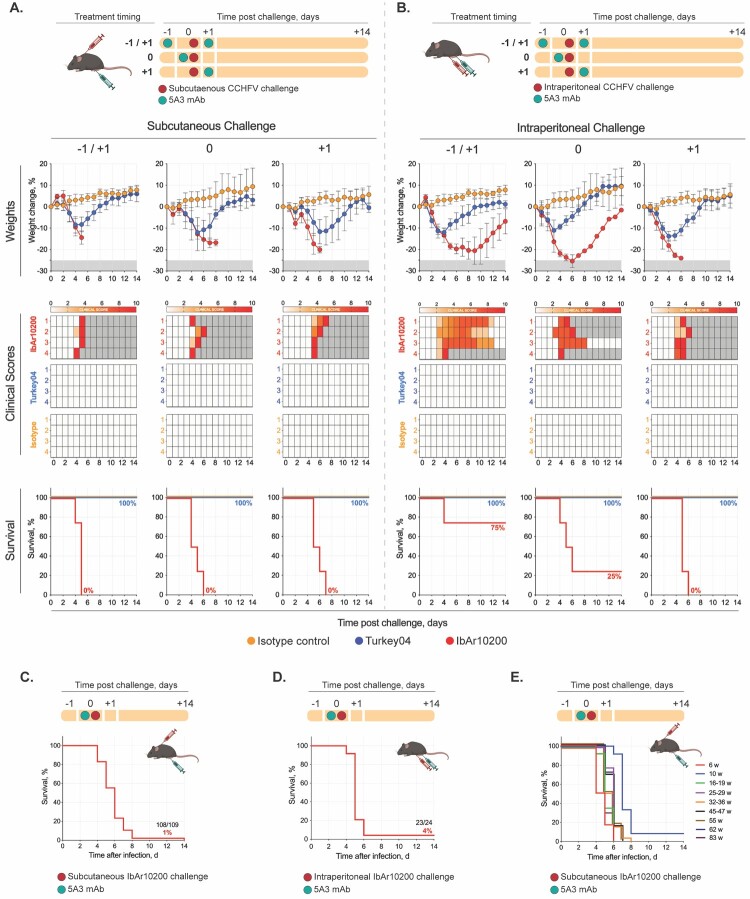
Lethal outcome in transiently immunosuppressed mice is dependent on CCHFV strain. In a pilot study, groups of C57BL/6J mice (4/group, mixed male [animal # 1–2] and female [animal # 3–4], 6 weeks of age) were infected either **(A)** subcutaneously (SC) or **(B)** intraperitoneally (IP) with 100 TCID_50_ of CCHFV strain IbAr10200 (red) or Turkey04 (blue). Mice received anti-IFNAR1 monoclonal antibody (mAb) 5A3 via IP injection on the following days post infection (dpi): −1/+1 (2.0 mg/0.5 mg), 0 (2.5 mg), or +1 (2.5 mg). Control animals (4/group; mixed male and female) were included for each experimental group (each challenge route [SC and IP] and mAb timing group [−1/+1, 0, +1 dpi]) and received an isotype control (IgG1) mAb rather than 5A3 (orange). Control animals were challenged with Turkey04. After challenge, weight loss (% change from baseline) and clinical scores (see Methods) were assessed daily for each animal until reaching euthanasia criteria (see Methods) or study completion (14 dpi). Individual dots and connecting lines represent the daily mean and error bars represent the standard deviation (SD). **(C–D)** Subsequent studies were performed using 0 dpi as the designated timing for 5A3 delivery (mixed male and female C57BL/6J mice, ages ranging from 6 to 83 weeks), resulting in high rates of lethality using both **(C)** SC and **(D)** IP routes of challenge. **(E)** Age-stratified cohorts of C57BL/6J mice at 6 (*n* = 12), 10 (*n* = 12), 16–19 (*n* = 14), 25–29 (*n* = 14), 32–36 (*n* = 17), 45–47 (*n* = 13), 55 (*n* = 6), 62 (*n* = 11), or 83 (*n* = 10) weeks of age were challenged (SC) with 100 TCID_50_ CCHFV IbAr10200 and 2.5 mg mAb 5A3 (0 dpi) and followed until meeting euthanasia criteria.

The CCHF IS model results in lethal disease after administration of mAb 5A3 (2.5 mg) as a split dose on −1/+1 days post infection (dpi) or a single dose +1 dpi using CCHFV strains IbAr10200, Afg09-2990, and Turkey-Kelkit06. [17,21,22]. These methods require 2–3 days of sequential anaesthesia to administer the mAb and challenge virus, and repeated anaesthesia can be linked to both adverse health effects and alterations to baseline immune responses in mice [23,24]. Recent work has demonstrated that uniform lethality can be achieved using a single dose of mAb 5A3 at 0 dpi, concurrent with delivery of the challenge virus, using the challenge strain UG3010 [25,26]. Here, we investigated whether infection with strain IbAr10200 could also result in lethal disease while limiting anaesthesia to a single event by administering 5A3 at 0 dpi. In a pilot study, groups of C57BL/6J mice (4/group; mixed male and female, 6 weeks of age) were infected either subcutaneously (SC; 100 µL) or intraperitoneally (IP; 200 µL) with 100 TCID_50_ of CCHFV strain IbAr10200. Subsets of mice received mAb 5A3 (IP; 400 µL) at one of the following timepoints: −1/+1 (2.0 mg/0.5 mg), 0 (2.5 mg), or +1 (2.5 mg) dpi. All animals were monitored daily for weight loss and signs of clinical disease until study endpoint 14 dpi. We found that 5A3 administration 0 dpi successfully resulted in lethal outcome in 7 of 8 mice (Figure 1(A–B)). The single survivor (IP challenge group) developed severe disease including weight loss and clinical signs including hunched posture, rough coat, and hypoactivity but recovered by 14 dpi (Figure 1(B)). Our pilot study also investigated parameters that have been documented to affect clinical outcome in mouse models of CCHF, including route of challenge and mouse sex [15,27,28]. We observed some differences in lethality based on route of inoculation with CCHFV IbAr10200. Lethality after IP inoculation varied and was 25%, 75%, and 100% when 5A3 was delivered on −1/+1, 0, and +1 dpi, respectively. All mice including survivors developed significant clinical signs indicating severe disease in the IP group. SC inoculation was uniformly lethal regardless of 5A3 timing. Time to death was similar between inoculation routes ranging from 4 to 6 days (IP) and 4–8 days (SC) (Figure 1(A–B)). Importantly, we did not find any notable differences based on mouse sex in the model; SC inoculation was 100% lethal in males and females, and IP inoculation was 83% lethal in both males and females.

Subsequent larger cohort studies using challenge strain IbAr10200 and 0 dpi as the designated timing for 5A3 delivery confirmed the utility of this approach resulting in lethality using both SC (99% lethal, *n* = 109 mice, ages ranging from 6 to 83 weeks) (Figure 1(C)) and IP (96% lethal, *n* = 24 mice, ages ranging from 6 to 10 weeks) (Figure 1(D)) routes of challenge. Using this approach, we investigated the effect of mouse age on lethality. This factor is important in the context of vaccine durability studies in which mice are vaccinated and challenged weeks or months later. In this study, C57BL/6J mice were challenged (SC) with 100 TCID_50_ CCHFV IbAr10200 and 2.5 mg mAb 5A3 (0 dpi) at 6 weeks and 10, 16–19, 25–29, 32–36, 45–47, 55, 62, and 83 weeks of age (Figure 1(E)). The IS model maintained lethality regardless of mouse age; across all age cohorts time to death ranged from 4 to 8 dpi (Figure 1(E)).

Pilot studies were performed using IbAr10200 (clade Africa 3), which is considered the prototype strain of CCHFV. We were interested in also evaluating strain Turkey04 (Turkey-200406546, clade Europe 1) because it is a lower passage human clinical isolate that causes uniformly lethal disease in IFNAR^-/-^ mice [29]. Groups of mice identical to those described in the pilot study were infected with CCHFV Turkey04 and all animals were monitored daily for weight loss and signs of clinical disease until study endpoint 14 dpi. Control animals infected with Turkey04 (4/group; mixed male and female) were also included for each experimental group (each challenge route [SC and IP] and mAb timing group [–1/+1, 0, +1 dpi]). These animals received an isotype control (IgG1) mAb at equivalent dosing and volume rather than 5A3. Unexpectedly, mice infected with Turkey04 transiently lost weight (10 to 15% of baseline on average), but showed no other signs of clinical disease, and all survived infection (Figure 1(A–B)). These results were consistent regardless of mAb timing, route of challenge, or sex of the animal (Figure 1(A–B)). A single animal in the 0 dpi/SC challenge group lost over 20% body weight, resulting in a clinical score of 7 (Figure 1(A)); however, this animal had no other signs of disease. Comparisons of IbAr10200- and Turkey04- infected mice show that weight loss in all animals began 1 to 2 dpi and followed a similar trajectory up to, on average, 4 dpi, when their clinical course diverged. Turkey04-infected animals regained weight and fully recovered by 8 to 12 dpi. IbAr10200-infected animals continued to lose weight and succumbed to infection 4 to 8 dpi (Figure 1(A–B)).

### Divergent clinical outcomes are not the result of differences in viral load or tissue tropism

To investigate the role that viral replication and tropism play in the divergent outcomes between strains, we conducted a serial sacrifice study where 6-week-old C57BL/6J mice (*n* = 4/cohort; mixed male and female) were infected SC or IP with CCFHV strain IbAr10200 or Turkey04 (100 TCID_50_) and euthanized immediately prior to their divergence in clinical disease course, at 3 dpi. Identical cohorts of Turkey04-infected animals (*n* = 4/cohort) were also euthanized 7 dpi to compare to terminal (4 to 8 dpi) IbAr10200-infected animals, and 14 dpi. Subsets of mice received mAb 5A3 via IP injection on one of the following dpi: −1/+1 (2.0 mg/0.5 mg), 0 (2.5 mg), or +1 (2.5 mg). Control animals (2/group; mixed male and female) in each experimental group received an isotype control (IgG1) antibody at equivalent dosing and volume as mice that received 5A3, and were challenged with Turkey04.

**Figure 2.**
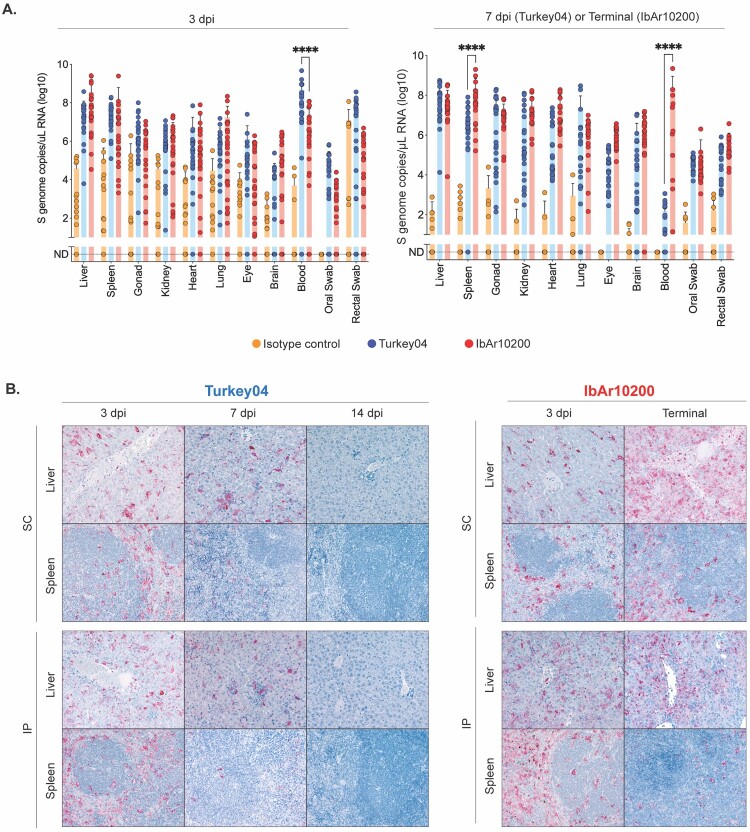
Divergent clinical outcomes are not the result of differences in viral load. Tissues including liver, spleen, ovary/testis (gonad), kidney, lung, heart, eye, brain, and whole blood, as well as oral and rectal swabs, were collected from all mice at the time of euthanasia at 3, 7, or 14 dpi (Turkey04) or 3 dpi and terminal timepoint (5–6 dpi; IbAr10200). Isotype control animals were challenged with Turkey04. **(A)** Viral RNA (vRNA) was isolated and quantified via RT-qPCR using primers/probes specific for the CCHFV S gene segment. Bars indicate the mean and error bars the standard deviation (SD). Statistics were calculated using two-way ANOVA with Tukey’s multiple comparison test; **** *p* < 0.0001. Only significant results are reported. **(B)** Immunohistochemistry (IHC) for CCHFV was performed using anti-CCHFV NP antibody with Fast Red chromogen. CCHFV antigen (red) amount and distribution is similar in liver and spleen tissues from animals infected with Turkey04 and IbAR10200 at 3 dpi but increases in terminal IbAr10200-infected tissues while decreasing in Turkey04-infected tissues at 7 dpi, and becoming undetectable by 14 dpi. Original magnifications: ×40 (liver); ×20 (spleen).

**Figure 3.**
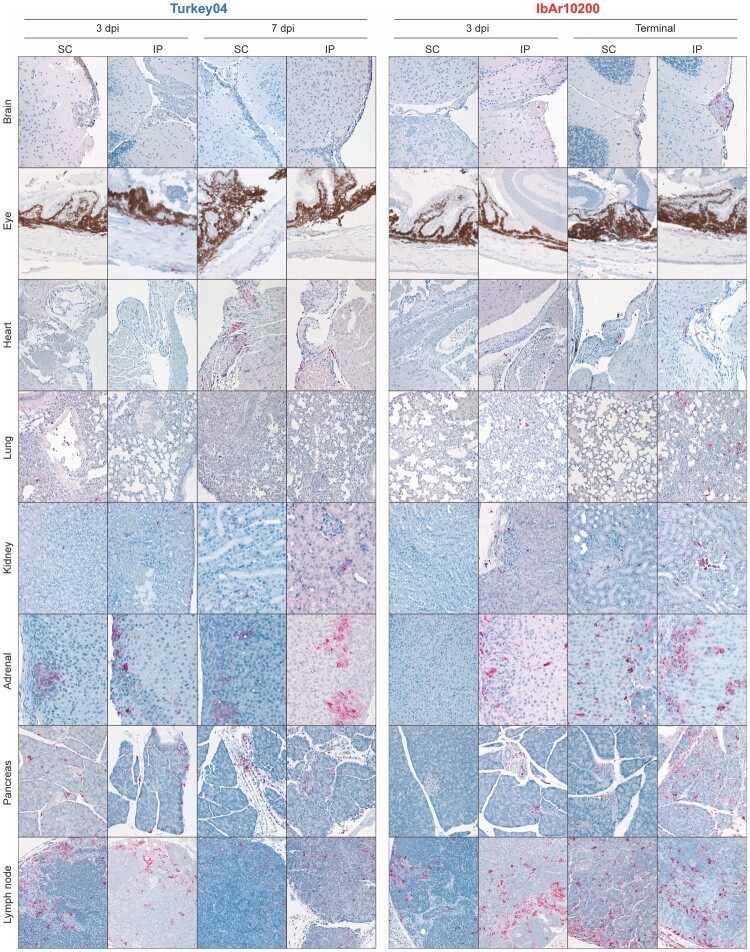
Divergent clinical outcomes are not the result of differences in tissue tropism. Tissues including brain, eye, heart, lung, kidney, adrenal gland, pancreas, and lymph node were collected from all mice at the time of euthanasia at 3, 7, or 14 dpi (Turkey04) or 3 dpi and terminal timepoint (5–6 dpi; IbAr10200). Immunohistochemistry (IHC) for CCHFV was performed using anti-CCHFV NP antibody with Fast Red chromogen within the brain, eye, heart, lung, kidney, adrenal gland, pancreas, and lymph node. CCHFV antigen (red) amount and tissue distribution are similar in animals infected with Turkey04 and IbAR10200. CCHFV antigen (red) is seen primarily within endothelial cells, intravascular leukocytes, and tissue macrophages, with either inoculation route and at both timepoints. Original magnifications: ×200.

The main target organs of CCHFV replication in both humans and mice are liver and spleen [30,31]. To comprehensively investigate differences in virus dissemination and tropism, we looked at these, as well as additional tissues not previously characterized in the IS model. Quantification of viral RNA (vRNA) via RT-qPCR and histopathology with detection of viral antigen via immunohistochemistry (IHC) were performed in the following tissues: liver, spleen, reproductive tissue, kidney, heart, lung, eye, brain, and blood. Pancreas, adrenal gland, and lymph nodes were assessed by histopathology and IHC only; blood was assessed by RT-qPCR only. Early after infection, at 3 dpi, we were surprised to find that levels of vRNA in 8 of 9 tissues were not significantly different between Turkey04-infected survivors and IbAr10200-infected non-survivors (Figure 2(A)). Interestingly, whole blood vRNA was significantly higher in Turkey04-infected survivors at this timepoint (Figure 2(A)). These data were supported by IHC, which showed comparable levels of viral antigen in major target organs (liver and spleen) (Figure 2(B)) and other tissues at 3 dpi (Figure 3).

Later during infection at 7 dpi, vRNA from surviving Turkey04-infected animals remained high overall and was only statistically lower in the blood and spleen compared with terminal (4–8 dpi) IbAr10200-infected animals (Figure 2(A)). At 14 dpi vRNA in recovered Turkey04-infected mice remained detectable at lower levels (Supplementary Figure 1). Viral antigen decreased in the liver and spleen of Turkey04-infected survivors from 3 to 7 dpi and was undetectable by 14 dpi. Conversely, viral antigen in IbAr10200-infected mice remained the same or increased in liver and spleen from 3 dpi to terminal timepoint (Figure 2(B)). In livers, staining for both viruses was observed primarily in hepatocytes, Kupffer cells, endothelial cells, and intravascular leukocytes, including those morphologically compatible with monocytes; in spleens staining was localized to mononuclear phagocytic cells, interpreted as macrophages and dendritic cells (Figure 2(B)). In other tissues, staining was primarily seen within endothelial cells, intravascular leukocytes, and tissue macrophages, and was also seen in adrenal epithelial cells and pancreatic interstitium and occasionally in the periphery of islets (Figure 3). Antigen distribution was consistent with what has been reported previously after CCHFV IbAr10200 infection in IFNAR^-/-^ mice [32].

Oral and rectal swabs were also collected to evaluate levels of vRNA (Figure 2(A)) and infectious virus (Supplementary Figure 2) in mucosal specimens. At 3 dpi, levels of vRNA in swab samples were not significantly different between Turkey04-infected survivors and IbAr10200-infected non-survivors (Figure 2(A)). Additional oral and rectal swab samples were collected during subsequent studies where mice were infected (SC) with either CCHFV Turkey04 or IbAr10200 (5A3 administered 0 dpi) and euthanized 3, 7, or 14 dpi (Turkey04) or 3 dpi and terminal timepoint (IbAr10200). Aggregate data from these studies show, on average, higher levels of vRNA in the oral and rectal swabs of surviving Turkey04-infected mice at 3 dpi (Supplementary Figure 3).

Overall, these data indicate that levels of viral replication, dissemination, and tissue tropisms are comparable between survivors and non-survivors immediately prior to divergence in clinical disease course, at 3 dpi, and that other factors are likely associated with the differences in outcomes.

### Lethal outcome with Ibar10200 is associated with progressive liver and spleen pathology

**Figure 4.**
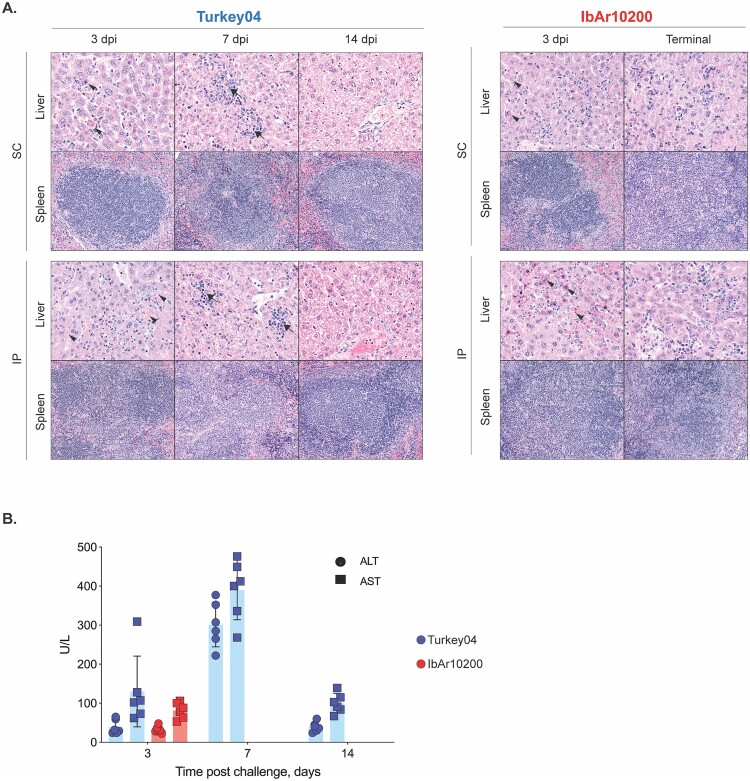
Lethal outcome with IbaR10200 is associated with progressive liver and spleen pathology. **(A)** Haematoxylin-eosin-stained liver and spleen from mice that were inoculated with mAb 5A3 and CCHFV SC or IP on day 0 and sacrificed 3, 7, or 14 dpi (Turkey04), or 3 dpi and at terminal timepoints (5–6 dpi; IbAr10200). Livers from both Turkey04 and IbAr10200-inoculated mice showed similar findings, with moderate hepatocellular necrosis (arrowheads) and neutrophilic inflammation at 3 dpi. At 7 dpi, livers from Turkey04-infected survivors showed decreased necrosis and increased inflammation (arrows), with addition of mononuclear infiltrates, and by 14 dpi, livers were normal. In contrast, at terminal timepoints, IbAr10200-infected livers showed continued progression and severity of changes. Spleens showed mild to moderate reactivity, with expansion of plasma cells and few macrophages and neutrophils in red pulp at 3 dpi in Turkey04-infected survivors. Lymphocyte necrosis/apoptosis was mild and more prominent in IP-inoculated animals. At 7 dpi, spleens from Turkey04-infected survivors showed marked lymphoid reactivity, with abundant plasma cells without lymphocyte necrosis/apoptosis. By 14 dpi, reactivity was present, but with fewer plasma cells compared to 7 dpi. For IbAr10200-inoculated animals, 3 dpi spleens were similar to spleens of Turkey04-infected animals at the same timepoint, but with more severe lymphoid reactivity and more prominent lymphocyte apoptosis/necrosis, especially in IP-inoculated animals. In terminal spleens of IbAr10200-infected animals, these changes were sustained, with more pronounced lymphoid necrosis/apoptosis also in SC-inoculated animals. Original magnifications: ×20 (spleen); ×40 (liver). **(B)** Liver enzymes (ALT, alanine aminotransferase; AST, aspartate aminotransferase) from each animal were assessed using whole blood (lithium heparin) at 3 (IbAr10200 and Turkey04), 7, or 14 (Turkey04 only) dpi. Bars indicate the mean and error bars the standard deviation (SD). Statistics were calculated using multiple t-tests (Mann–Whitney); no significant differences between IbAr10200 and Turkey04 were found at 3 dpi.

Given the similarities in early tissue distribution and viral loads of both IbAr10200 and Turkey04, we next investigated whether differences in tissue pathology and inflammatory response were associated with differences in outcomes. Histopathologic evaluation of livers showed similar findings at 3 dpi, regardless of virus strain or inoculation route. These included moderate single cell and confluent hepatocellular necrosis, prominent acute, neutrophilic inflammation, and intravascular leukocytosis (Figure 4(A)); occasional intravascular thrombi were also seen. Liver enzyme values (aspartate aminotransferase [AST] and alanine aminotransferase [ALT]) were also assessed at 3 dpi, confirming no difference in the extent of acute liver damage between survivors and non-survivors early after infection (Figure 4(B)). Livers of Turkey04-infected survivors showed less necrosis and more inflammation at 7 dpi, with a transition from predominately neutrophils to predominately mononuclear cells (macrophages and lymphocytes). By 14 dpi, these livers were essentially normal. Conversely, in livers of terminal (4–6 dpi) IbAr10200-infected mice, changes were similar to, but more severe than seen at 3 dpi, with progressive necrosis and acute inflammation with notably less prominent influx of lymphocytes (Figure 4(A)).

Spleens of Turkey04- and IbAR10200-infected mice showed subtle differences in histopathology, even at 3 dpi. Both had red pulp infiltration by macrophages and neutrophils, and lymphoid reactivity with plasma cell expansion and scattered lymphocyte necrosis/apoptosis, the latter of which was more prominent in spleens of IbAr10200-infected mice, and in IP-inoculated animals for both strains (Figure 4(A)). At 7 dpi, spleens of Turkey04-infected survivors showed marked lymphoid reactivity with abundant plasma cells, but without lymphocyte necrosis/apoptosis, and by 14 dpi, reactivity was present but with fewer plasma cells than 7 dpi. However, in spleens of terminal (4–6 dpi) IbAR10200-infected mice, lymphoid reactivity and plasma cell expansion were marked and accompanied by prominent lymphocyte necrosis/apoptosis regardless of inoculation route (Figure 4(A)).

### Early inflammation associated with monocyte/macrophage recruitment and activation corresponds with lethal outcome

**Figure 5.**
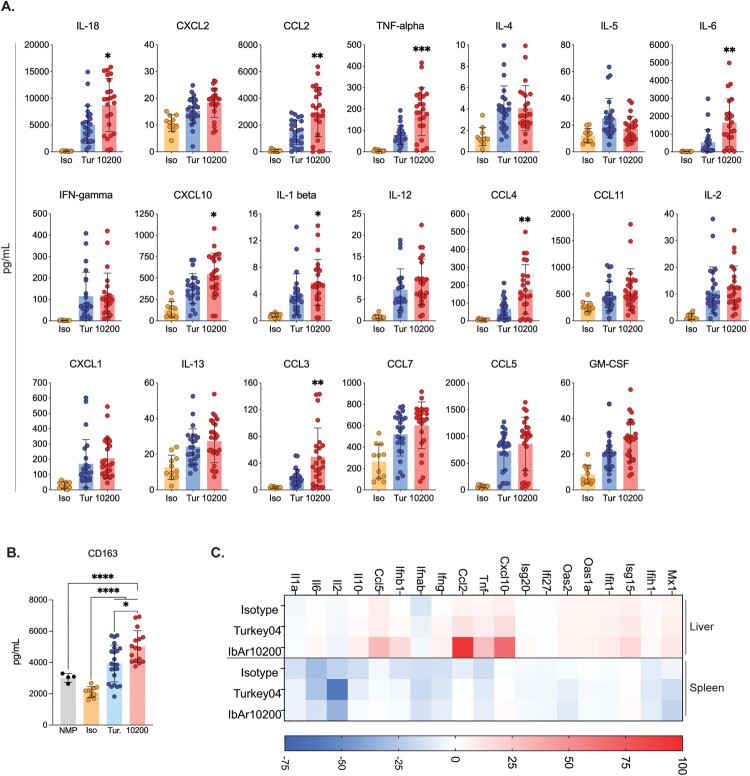
Inflammation associated with monocyte/macrophage recruitment and activation corresponds with lethal outcome. **(A)** Cytokine/chemokine responses in all mice euthanized 3 dpi were analysed using the ProcartaPlex Mouse Th1/Th2 Cytokine and Chemokine panel and 25 µL mouse plasma. Statistics compare IbAr10200- to Turkey04-infected animals. **(B)** Plasma CD163 levels were measured in all animals euthanized 3 dpi as well as from (*n* = 4) naive age-matched C57BL/6J mice to determine baseline levels in healthy mice. **(C)** Gene expression profiles in liver and spleen of all mice euthanized 3 dpi were analysed via RT-qPCR and are reported as fold change compared to tissue from age-matched uninfected C57BL/6J control animals. Isotype control animals were infected with CCHFV Turkey04. Bars indicate the mean and error bars the standard deviation (SD). Statistics were calculated using multiple *t*-tests (Mann–Whitney); ** *p* < 0.01; *** *p* < 0.001; **** *p* < 0.0001. Only significant results are reported. Iso, isotype control; Tur, CCHFV Turkey04; 10200, CCHFV IbAr10200; NMP, normal mouse plasma.

Plasma cytokines and chemokines were analysed from all mice euthanized at 3 dpi. In non-surviving IbAr10200-infected mice compared to surviving Turkey04-infected mice, we found TNF-alpha (*p* < 0.001), IL-6, CCL2 (monocyte chemoattractant protein 1 [MCP-1]), CCL3 (macrophage inflammatory protein 1-alpha [MIP-1 alpha]), and CCL4 (macrophage inflammatory protein 1-beta [MIP-1 beta]) (*p* < 0.01) to be most significantly elevated, followed by IL-1 beta, CXCL10 (macrophage inflammatory protein 2-alpha [MIP-2 alpha]), and IL-18 (*p* < 0.05), indicating a higher level of systemic inflammation in lethal infection (Figure 5(A)).

In particular, a large proportion of the upregulated analytes (8 of 20) that correspond with lethality are associated with macrophage activation and/or chemoattraction [33]. Macrophage activation syndrome (MAS; also referred to as hemophagocytic lymphohistiocytosis syndrome [HLS]) is a rheumatologic disorder that features cytokine storm (hypercytokinemia) driven by activated macrophage cell populations. This syndrome has been recognized during severe Ebola virus disease (EVD) [34,35] and has also been described during human cases of CCHF [36–38]. Given our finding of macrophage-associated hypercytokinemia, we searched for additional evidence of MAS in IbAr10200- compared to Turkey04-infected animals by evaluating plasma levels of macrophage activation marker CD163. We found that IbAr10200-infected mice had significantly elevated plasma CD163 compared to uninfected normal C57BL/6 mice and Turkey04-infected mice (Figure 5(B)) at 3 dpi, supporting the finding that macrophage-associated inflammation early after infection corresponds with lethal outcome.

We next investigated whether an anti-folate receptor beta IgG2a monoclonal antibody (mAb) would reduce inflammation in CCHFV IbAr10200 infected mice. This mAb can target and reduce activated macrophages in the context of some chronic diseases but has not been explored in the context of viral infection. In our hands, this approach did not reduce inflammation nor improve outcome related to CCHFV infection (Supplementary Figure 4).

Lastly, we looked at gene expression profiles in both the liver and spleen via RT-qPCR at 3 dpi. Gene expression data are reported as fold change compared to tissue from age-matched uninfected C57BL/6J control animals (Figure 5(C)). In the liver, CCL2 expression was over 80-fold higher in non-surviving IbAr10200-infected animals compared to surviving Turkey04-infected animals (Figure 5(C)). Additionally, CXCL10, CCL5, and TNF were all over 20-fold higher in these animals. Interestingly, in the spleen, gene expression profiles of survivors (Turkey04) and nonsurvivors (IbAr10200) were similar or slightly downregulated in surviving Turkey-04-infected animals (Figure 5(C)). For example, IL-6 and IL-10 expression levels in the spleen were 2- and 3-fold lower, respectively, in surviving Turkey04-infected animals (Figure 5(C)). The most pronounced difference was in IL-2 expression which was 25-fold lower in surviving Turkey04-infected animals than in IbAr10200-infected non-survivors (Figure 5(C)).

### Mouse macrophages are more permissive to IbAr10200 infection in vitro

**Figure 6.**
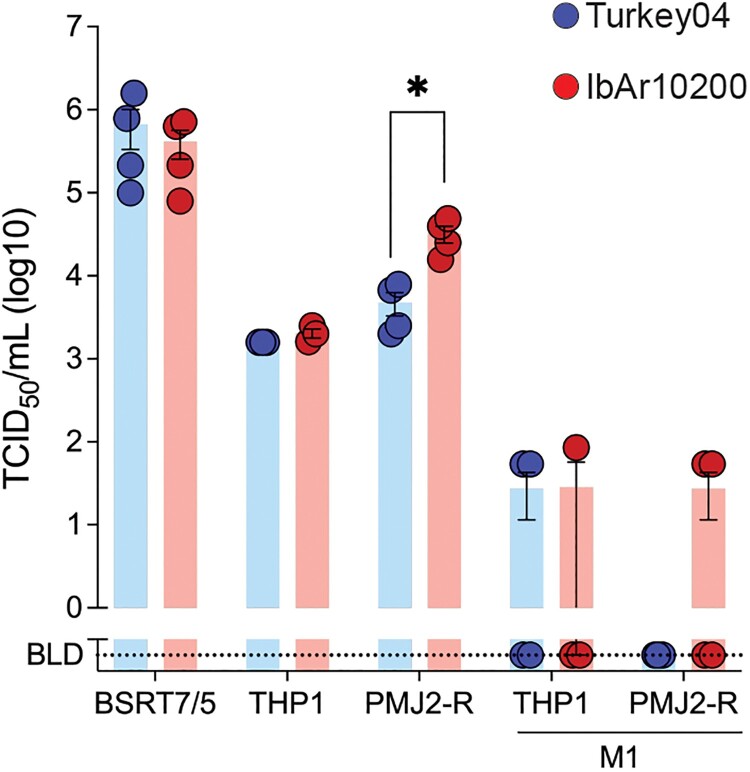
Mouse macrophages are more permissive to IbAr10200 infection in vitro. CCHFV IbAr10200 and Turkey04 viruses were titered via immunofluorescent TCID_50_ assays in BSRT7/5 (baby hamster kidney cell stably expressing T7 polymerase), PMJ2-R (C57BL/6J peritoneal macrophage), and THP-1 (human monocyte) cells. Prior to titering, THP-1 cells were differentiated into macrophages by incubating with 5 ng/mL PMA for 24 h. Titers were also determined on polarized PMJ2-R and THP-1 cells (classical, M1 phenotype) by incubating with 250 ng/mL lipopolysaccharide (LPS) and 20 ng/mL mouse or human IFN-gamma for 48 h. Bars indicate the mean and error bars the standard deviation (SD). Statistics were calculated using multiple *t*-tests (Mann–Whitney); * *p* < 0.05; only significant results are reported.

While we did not observe differences in viral tropism at the tissue level between IbAr10200- and Turkey04-infected mice at 3 dpi, we were interested in investigating tropism for monocytes/macrophages after finding macrophage-associated cytokines/chemokines to be elevated during lethal CCHFV infection. These cells have also been documented to be the main targets of CCHFV infection in humans and mice [30,32,39]. We evaluated the infectivity of the two viruses in various cell lines including BSRT7/5 cells because these were used to quantify the *in vivo* challenge inoculum, as well as human and C57BL/6 mouse monocyte/macrophage cell lines (THP-1 and PMJ2-R, respectively) (Figure 6). We found mouse macrophage (PMJ2-R) permissiveness to be 10-fold higher for CCHFV strain IbAr10200 than Turkey04 (*p* = 0.0286), but this was not the case for human-derived macrophages (THP-1) (Figure 6). Activated macrophages from both humans and mice (M1 phenotype) appeared to be refractory to infection with either virus. Infectivity in BSRT7/5 cells was not significantly different between the two viruses. These data are suggestive that the efficiency of viral entry into monocytes/macrophages could play a role in strain-dependent pathogenesis of CCHFV, however additional studies would be required to confirm the role of these cells.

## Discussion

In this report, we advance mouse models for CCHF by providing key data on experimental approaches to the transiently immunosuppressed (IS) mouse model (time of mAb 5A3 administration, challenge route, sex and age effects, virus strain effects). Using these novel approaches to IS, we also advance knowledge on CCHF biomarkers by performing detailed investigations into factors underlying lethal and non-lethal infection, finding that markers of inflammation, independent of viral replication, are associated with terminal outcomes.

The IS model remained lethal when mAb 5A3 was administered at the time of challenge (0 dpi) with strain IbAr10200. This and previously reported data using strain UG3010 [25,26] demonstrate an approach that is safer and logistically preferred for work in high containment, reducing the use of needles and need for multiple anaesthesia events that can confound results [23,24]. To date, the IS model has only been reported using IP challenge. SC injection is lethal in IFNAR^-/-^ mice using multiple virus strains [29] and better mimics the predominant natural route of CCHFV infection (tick bite) but can result in attenuation in certain animal models [27,28]. We found that SC challenge with IbAr10200 resulted in a similar time to death and mortality as the IP route. Also, while sex-based differences in disease severity have recently been reported for the mouse-adapted CCHFV mouse model [15], we confirmed that the IS model did not demonstrate a sex-bias (comparable to infection in IFNAR^-/-^ mice). This further supports the utility of the IS model for evaluation of vaccine or therapeutic candidates. Finally, we demonstrate the value of the lethal IS model for long-term studies (*e.g.* vaccine durability), as aged mice up to 83 weeks old remain susceptible to lethal infection.

Several strains of CCHFV cause mild or subclinical infection in IFNAR^-/-^ mice [29]. Strain-dependent pathogenesis has also been described in the context of the IS model; Afg09-2990 (clade Asia 1) was uniformly lethal while Kosovo Hoti (clade Europe 1) was not [12]. Despite several previous studies in IFNAR^-/-^ mice causing lethal outcome using the same Turkey04 stock, here in the IS model it resulted in non-lethal disease characterized by transient weight loss while IbAr10200 remained uniformly lethal. Identification of a CCHF survivor model using Turkey04 has important application for future studies investigating long-term sequelae, viral persistence, and recrudescence after CCHFV infection. Further, as we demonstrate here, contrasting lethal and survivor mouse models can provide insight into factors contributing to CCHF pathogenesis.

We found that immediately before the divergence in clinical course between IbAr10200- and Turkey04-infected mice, there was no difference in viral replication or tissue dissemination between strains. The earliest difference we identified were significantly elevated levels of inflammation associated with macrophage/monocyte activation and recruitment in the plasma and livers of IbAr10200-infected non-survivors. While this inflammation (upregulation of TNF alpha, IL-6, CCL2, CCL3, CCL4, IL-1 beta, CXCL10, and IL-18) cannot exclusively be attributed to activated macrophages as this has not been definitively studied during CCHFV infection, there is clear evidence that these cells are often predominant contributors to their production [33].

Macrophage activation syndrome (MAS), also referred to as hemophagocytic lymphohistiocytosis syndrome (HLS), has been described in severe human cases of CCHF [36–38] and other viral haemorrhagic fevers (VHFs) such as Ebola virus disease (EVD) [34]. It features a similar profile of hypercytokinemia, as well as fever, liver dysfunction, and coagulopathy. In humans, plasma levels of macrophage activation marker CD163 are considered an important indicator of MAS [34,35]. While rodent and human CD163 expression profiles differ and plasma CD163 is known to be lower in rodents than humans, activated rodent macrophages do release large numbers of CD163-positive exosomes during inflammatory diseases that can be detected in plasma, indicating activation of tissue resident macrophages [40,41]. Additionally, proinflammatory cytokines from activated T cells have also been proposed to contribute to pathogenesis during MAS [34,35]. In our study, we found plasma CD163 to be significantly elevated in non-surviving (IbAr10200-infected) mice. We also found 25-fold higher IL-2 expression in the spleens of non-surviving IbAr10200-infected animals. IL-2 is primarily produced by activated CD4^+^ and CD8^+^ T cells [42] and this data suggests a higher level of T cell activation in the spleens of non-survivors early after infection. Altogether, these data support that a MAS-like process may be associated with fatal CCHF disease.

Differences in tissue pathology between survivors and non-survivors primarily become apparent later in infection, following differences in acute inflammation. Liver pathology suggests that survival is associated with an inflammatory response initially characterized by neutrophil and macrophage recruitment, which then transitions to lymphocytic infiltrates. In lethal cases, acute inflammation is sustained and a prominent shift toward lymphocyte recruitment is not observed. In the spleen, greater levels of inflammation were observed early after infection (3 dpi) in non-survivors. Interestingly, histopathology showed some degree of splenic reactivity with plasma cell expansion in all animals regardless of outcome, indicating intact functional lymphoid responses. However, lymphocyte necrosis/apoptosis was more pronounced in non-survivors, suggesting that adaptive immune responses may be hampered in these animals, and consistent with the absence of adaptive immune responses typically reported in fatal human cases of CCHF [43–45].

Later in the disease course and consistent with the histopathology findings, vRNA from Turkey04-infected mice (7 dpi) are significantly reduced in both the blood and spleen compared to terminal IbAr10200-infected mice (4-8 dpi), suggesting that after 3 dpi the Turkey04 strain was cleared more effectively from these tissues, potentially contributing to survival. Indeed, lower levels of viremia correspond with improved outcomes in human cases of CCHF [46–48]. Whether the viral clearance is due to better adaptive responses and/or stronger antiviral immune antagonism would need to be investigated. One limitation of these data is that we do not have serial data for Turkey04 to match each of the range of timepoints at which IbAr10200-infected animals met euthanasia criteria or succumbed to infection. However, we think the timepoints included in the analyses (IbAr10200 at 4-8 dpi versus Turkey04 at 7 dpi) represent a similar period in infection and are important in highlighting general trends in tissue pathology and virus replication kinetics later during infection. Overall, dynamic differences in the replication levels of the two strains after 3 dpi cannot be discounted, particularly in the spleen and blood, as contributing to the divergent virulence observed in the IS model.

A central feature of several VHFs is the infection of innate immune cells such as monocytes and macrophages, leading to a dysregulated inflammatory response [49,50]. Numerous studies in both humans and mice suggest a role for monocytes/macrophages in CCHFV pathogenesis. Both human [30] and mouse [32] studies indicate mononuclear phagocytes as the main cellular target of CCHFV infection, and it has been reported that strain virulence may positively correlate with entry efficiency into monocytes/macrophages [39]. Our data also demonstrate more effective entry of the virulent IbAr10200 strain into mouse macrophages *in vitro*, suggesting that this difference in tropism could play a role in disease outcome through early induction of proinflammatory responses. However, future studies would be needed to confirm this hypothesis, for example, macrophage depletion studies in mice.

Limitations associated with IFNAR^-/-^ mouse models are also present in IS models where a wide array of immune responses can be affected by the absence of IFNAR-I signalling at the time of virus challenge. These include alterations to the differentiation and signalling of antigen presenting cells, proliferation and survival of T cells, and activation and class switching of B cells [18]. For this reason, caution should be taken when interpreting results and their relevance to other model systems or humans. Despite similar limitations, the IS model system is also clearly distinct from IFNAR^-/-^ mouse models. CCHFV strain Hoti is lethal in IFNAR^-/-^ mice [27] but non-lethal in the IS model system when administered IP [12]. Studies using Dengue virus have also demonstrated uniform lethality using the same virus strain and challenge route in IFNAR^-/-^ mice but not the IS model [51]. These discrepancies are also evident with the Turkey04 strain reported here. One factor that may contribute to fundamental differences in the two model systems is that 5A3 mAb blockage will wane in the IS model (half-life approximately 5 days *in vivo*). Further, blockage may also not be absolute, and breakthrough or leak of IFN-I responses may impact viral pathogenesis [18]. Differences in pathogenesis between the two models using the same challenge strains/routes may point to enhanced sensitivities of specific virus strains to IFN-I responses; here, we could speculate that Turkey04 may be more sensitive to IFN-I responses than IbAr10200.

Golden *et al.* (2022) also reported strain-specific differences in the IS model using strains Afg09-2990 and Kosovo Hoti, the latter virus causing non-lethal disease associated with lower host inflammatory responses [12]. Interestingly, CCHFV Kosovo Hoti and Turkey04, both of which cause less pronounced proinflammatory immune responses and non-lethal disease in the IS model, are clade Europe 1 viruses with high sequence identity. This is in contrast with human data where high CCHF case fatality rates have been reported from the Kosovo region where Hoti was isolated (25.5%) [52] while lower rates are frequently reported from Turkey (5%) [53]. We therefore cannot discount the effect of viral passage history on differences in pathogenesis in the IS model, where Kosovo Hoti, Turkey04, and IbAR10200 have been passaged 0, 1, and 9 times, respectively, in suckling mouse brain [31]. Overall, further information is needed to pinpoint specific viral genetic determinants of pathogenesis for CCHFV. Instead, our primary focus is on the *in vivo* disease phenotype and what this might reveal about host factors underlying survival. The conclusion that inflammatory host responses are critical for CCHF outcome derived from independent mouse studies using different virus strains suggests a conserved mechanism of pathogenesis. Critically, it also aligns with findings from human cases in which mortality correlates with elevated levels of systemic inflammation [8–10], supporting the relevance of these mouse models for investigating pathogenesis.

Limited work has been done to investigate whether immunomodulatory therapeutics can protect against CCHF. Anti-TNF-alpha antibodies were shown to provide partial (50%) protection against lethal CCHFV infection in mice [12]. Corticosteroid treatment has also been anecdotally demonstrated to benefit severe human cases of CCHF [37,54,55] but did not provide protection from lethal disease in mice challenged with a mouse-adapted CCHFV [56]. Mice with MAS can be protected via anti-IFN-gamma treatment [57,58] but this was not the case during CCHFV infection in an IFNAR^-/-^ mouse model where IFN-gamma was necessary for survival [59]. In our study IFN-gamma was not associated with lethality, thus the mechanisms underlying CCHFV pathogenesis in our study may be distinct from other mouse models of MAS, but also distinct from those described in the IFNAR-/- mouse model [59]. We investigated whether an anti-folate receptor beta mAb that can promote clearance of activated macrophages during chronic disease could reduce inflammation during CCHFV IbAr10200 infection. In our hands, following previously described approaches in mice, this specific mAb did not reduce inflammation or increase survival. However, this approach requires functional natural killer (NK) cells for efficacy, and more data are required to understand if NK cells are hampered during CCHFV infection in mice. Overall, our data support additional research into therapeutics that can reduce inflammation, with a focus on approaches that have fewer off-target effects than corticosteroids but greater efficacy than reduction of a single cytokine/chemokine alone.

CCHF research, in particular animal model research, has long been limited by the requirement for immunosuppressed knockout strains to recapitulate lethal disease. Here, we provide critical advances in the use of transient immunosuppression to model CCHF in mice and demonstrate use of this model to identify indices associated with improved clinical outcomes. These studies indicate that early inflammatory responses after infection are the first indicators of, and contributors to, poor outcomes and should be prioritized in future therapeutic screening and development for CCHF.

## Supplementary Material

Supplementary Materials_IS Model.docx
