# Supplementary material for: Inflammation associated with monocyte/macrophage activation and recruitment corresponds with lethal outcome in a mouse model of Crimean-Congo haemorrhagic fever1
Source: Emerg Microbes Infect. 2024 Nov 8;13(1):2427782. doi: 10.1080/22221751.2024.2427782 (PMC11578417; doi:10.1080/22221751.2024.2427782)
Supplement: Supplementary Materials_IS Model.docx [file TEMI_A_2427782_SM4945.docx]

**Inflammation associated with monocyte/macrophage activation and recruitment corresponds with lethal outcome in a mouse model of Crimean-Congo hemorrhagic fever**

Teresa E. Sorvillo^1,2*^, Jana M. Ritter^3^, Stephen R. Welch^1^, JoAnn D. Coleman-McCray^1^, Katherine A. Davies^1,4^, Heather M. Hayes^3^, Scott D. Pegan^5^, Joel M. Montgomery^1^, Éric Bergeron^1^, Christina F. Spiropoulou^1^, Jessica R. Spengler^1^

**Affiliations:**

^1^Viral Special Pathogens Branch, Division of High Consequence Pathogens and Pathology, Centers for Disease Control and Prevention, 1600 Clifton Road, Atlanta, GA, USA

^2^Infectious Disease Department, CDC Foundation, Atlanta, GA, USA

^3^Infectious Diseases Pathology Branch, Division of High Consequence Pathogens and Pathology, Centers for Disease Control and Prevention, 1600 Clifton Road, Atlanta, GA, USA

^4^Zoonotic and Emerging Disease Research Unit, National Bio and Agro-Defense Facility, Agricultural Research Service, United States Department of Agriculture, Manhattan, Kansas, USA

**Supplementary Figure 1.** Viral RNA remains detectable in swabs and tissues from surviving CCHFV Turkey04-infected mice 14 days post infection.

**Supplementary Figure 2.** Infectious CCHFV can be detected from a subset of PCR positive rectal swabs collected at 3 dpi and terminal timepoints.

**Supplementary Figure 3.** Viral RNA is detectable in mucosal swabs from CCHFV-infected mice throughout the course of infection in both lethal and nonlethal models.

**Supplementary Figure 4.** Anti-folate receptor beta monoclonal antibody does not reduce inflammation in CCHFV IbAr10200-infected mice.

**
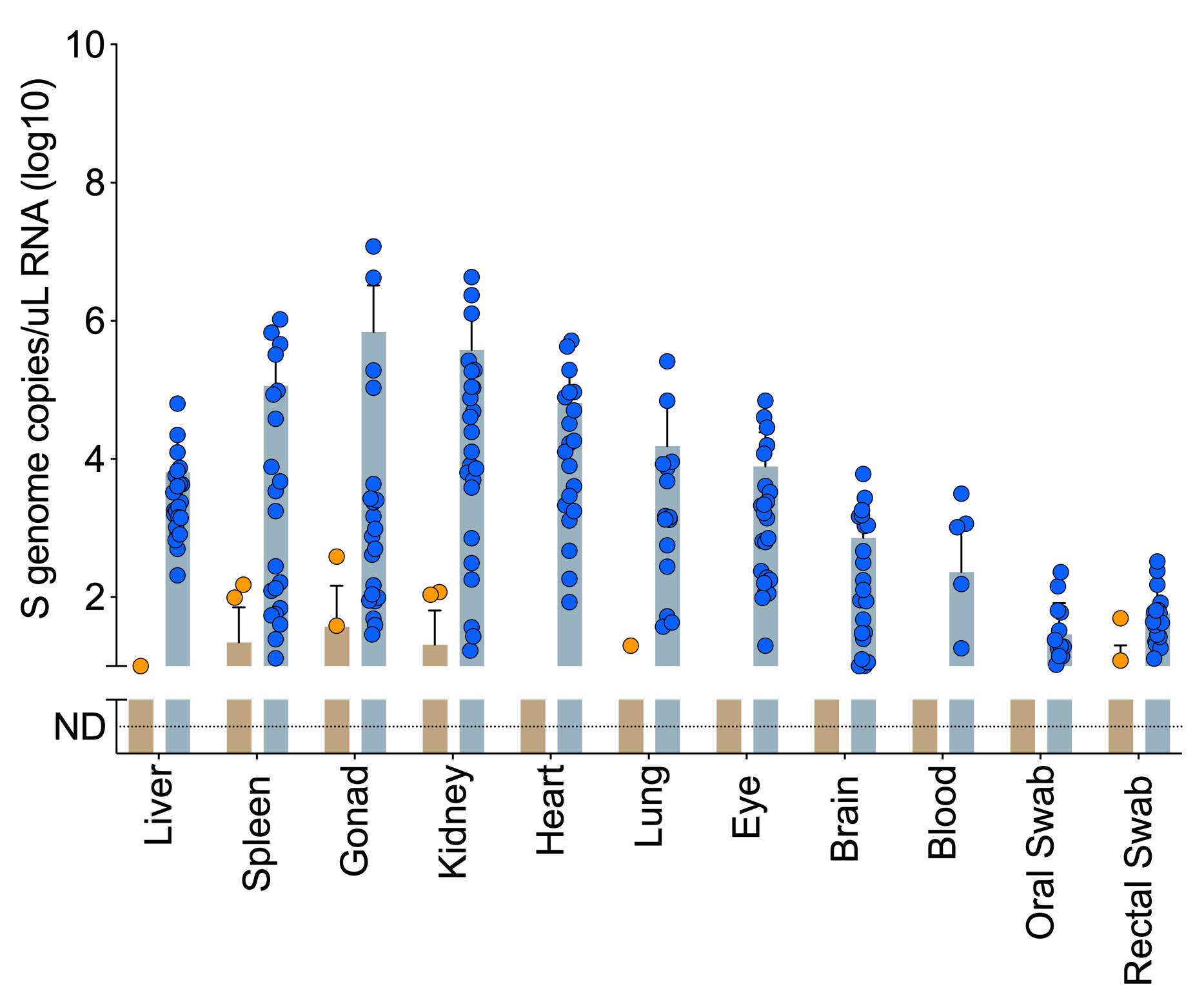
**

**Supplementary Figure 1. Viral RNA remains detectable in swabs and tissues from surviving CCHFV Turkey04-infected mice 14 days post infection.** C57BL/6J mice (4/group; mixed male and female, 6 weeks of age) were infected either subcutaneously (SC) or intraperitoneally (IP) with 100 TCID_50_ of CCHFV strain Turkey04 (blue). Subsets of mice were immunosuppressed through delivery of mAb 5A3 (IP) at one of the following timepoints: -1/+1 (2.0 mg/0.5 mg), 0 (2.5 mg), or +1 (2.5 mg) days post infection (dpi). Control animals (2/group; mixed male and female) in each experimental group received an isotype control (IgG1) antibody instead of 5A3 (green). All 5A3-treated animals transiently lost weight but showed no other signs of clinical disease and survived until the study endpoint of 14 days post infection (dpi). Tissues including liver, spleen, ovary/testis (gonad), kidney, heart, lung, eye, brain, and whole blood, as well as oral and rectal swabs, were collected from all mice at the time of euthanasia (14 dpi). Viral RNA (vRNA) was isolated and quantified via RT-qPCR using primers/probes specific for the CCHFV S gene segment. Individual animals are represented. Bars and error bars indicate the mean ± standard deviation (SD).

**
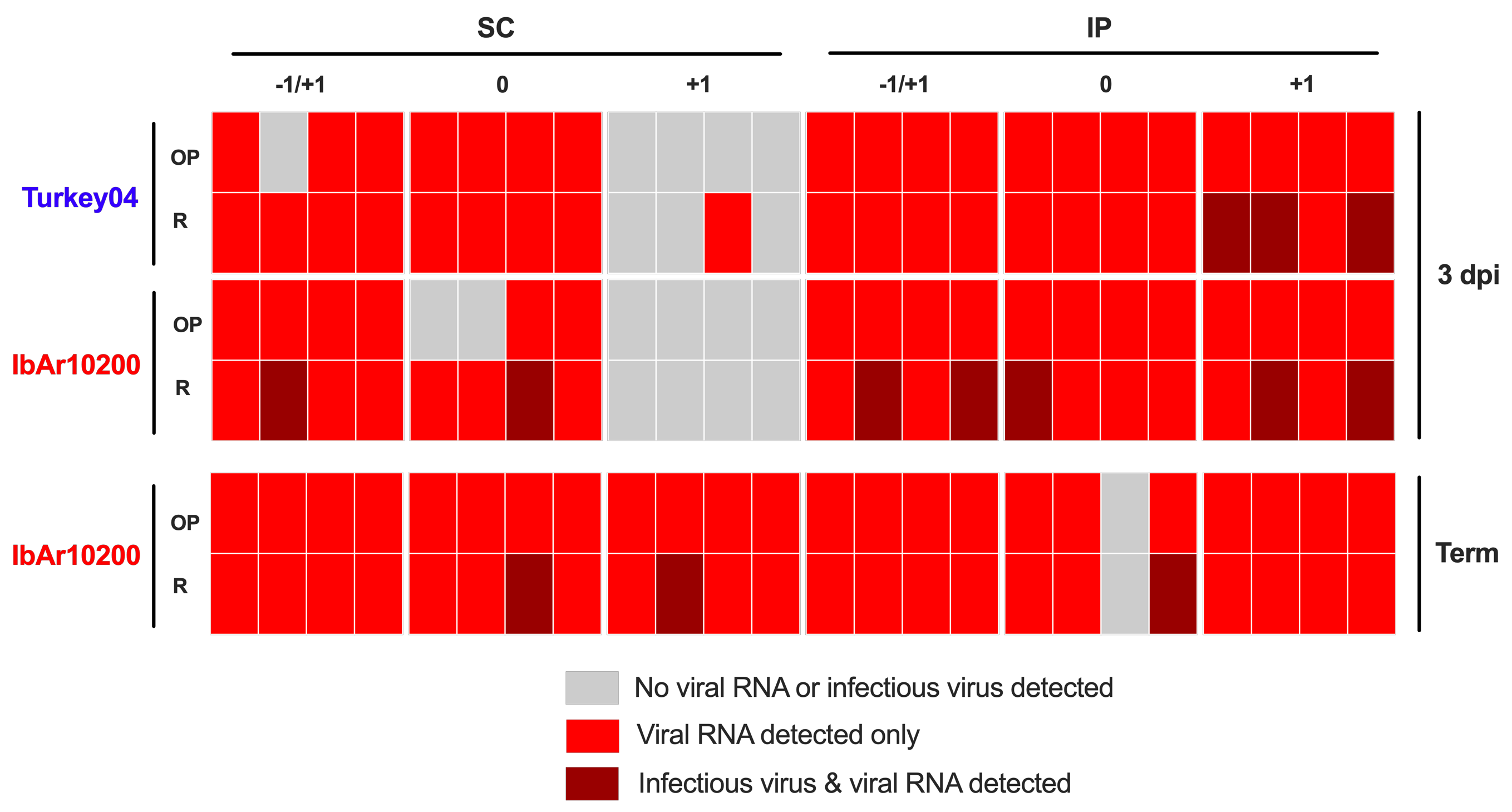
**

**Supplementary Figure 2. Infectious CCHFV can be detected from a subset of PCR positive rectal swabs collected at 3 dpi and terminal timepoints.** C57BL/6J mice (4/group; mixed male and female, 6 weeks of age) were infected either subcutaneously (SC) or intraperitoneally (IP) with 100 TCID_50_ of CCHFV strain Turkey04 or IbAr10200. Subsets of mice were immunosuppressed through delivery of mAb 5A3 (IP) at one of the following timepoints: -1/+1 (2.0 mg/0.5 mg), 0 (2.5 mg), or +1 (2.5 mg) days post infection (dpi). Oropharyngeal (OP) and rectal (R) swabs were collected for virus isolation from mice euthanized at 3 dpi (IbAr10200 and Turkey04) or terminal timepoints (IbAr10200 only). Virus isolation attempts were performed using BSR-T7/5 cells and samples were scored positive or negative based on the presence or absence of immunofluorescent foci. Positive samples were further analyzed via immunofluorescent TCID_50_ assay to quantify infectious virus. Infectious virus was detectable from a subset of rectal swabs but not oral swabs at 3 dpi (3 of 24, Turkey04; 7 of 24, IbAr10200) and terminal timepoints (3 of 24, IbAr10200). All positive samples were below the limit of detection for TCID_50_ quantification except one terminal CCHFV IbAr10200-infected animal (21.7 TCID_50_/mL). Each box represents data from an individual animal.

**
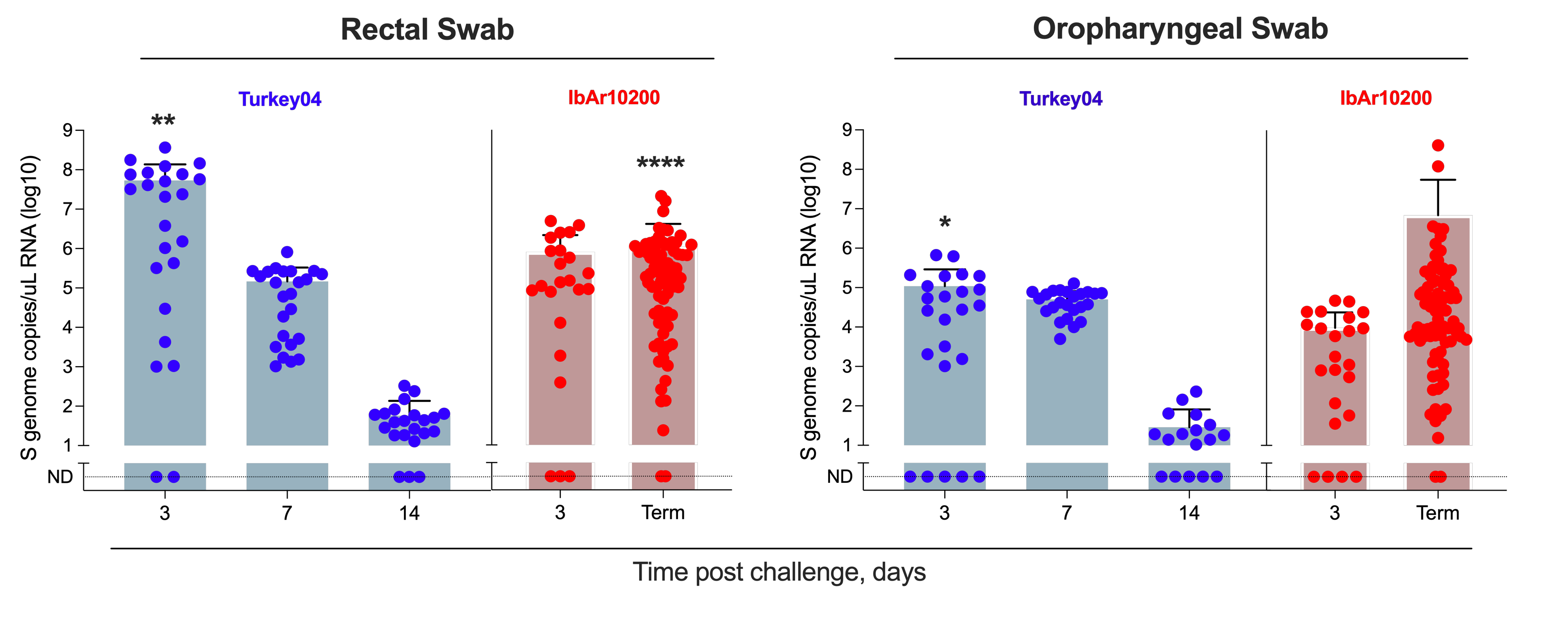
**

**Supplementary Figure 3. Viral RNA is detectable in mucosal swabs from CCHFV-infected mice throughout the course of infection in both lethal and nonlethal models.** C57BL/6J mice (mixed male and female, ages ranging from 6 to 55 weeks of age) were infected either subcutaneously (SC) or intraperitoneally (IP) with 100 TCID_50_ of CCHFV strain Turkey04 or IbAr10200. Mice were immunosuppressed through delivery of mAb 5A3 (IP) at one of the following timepoints: -1/+1 (2.0 mg/0.5 mg), 0 (2.5 mg), or +1 (2.5 mg) days post infection (dpi). Oropharyngeal and rectal swabs were collected from mice euthanized at 3, 7, and 14 dpi (Turkey04) or 3 dpi and terminal timepoint (IbAr10200). Viral RNA (vRNA) was isolated and quantified via RT-qPCR using primers/probes specific for the CCHFV S gene segment. Individual animals are represented. Bars and error bars indicate the mean ± standard deviation (SD). In Turkey04-infected mice, vRNA was detectable in 79% of oral swabs (19/24) and 91% of rectal swabs (22/24) at 3 dpi, 100% of both oral (24/24) and rectal (24/24) swabs at 7 dpi, in 79% of oral swabs (19/24) and 88% rectal swabs (21/24) at 14 dpi. In IbAr10200-infected mice, vRNA was detectable in 83% of oral swabs (20/24) and 88% of rectal swabs (21/24) at 3 dpi, and in 98% of both oral (78/80) and rectal swabs (78/80) at terminal timepoints. Statistical comparisons (multiple t-tests [Mann Whitney]; * p < 0.05; ** p < 0.01; **** p < 0.0001) were designed to compare virus strains at similar timepoints: 3 dpi, and 7 dpi (Turkey04) compared to terminal (IbAr10200). Higher levels of vRNA were detected in the oral and rectal swabs of Turkey04-infected mice at 3 dpi. At terminal timepoints IbAr10200-infected mice had higher levels of vRNA in rectal swabs compared to Turkey04-infected animals at 7 dpi.

**
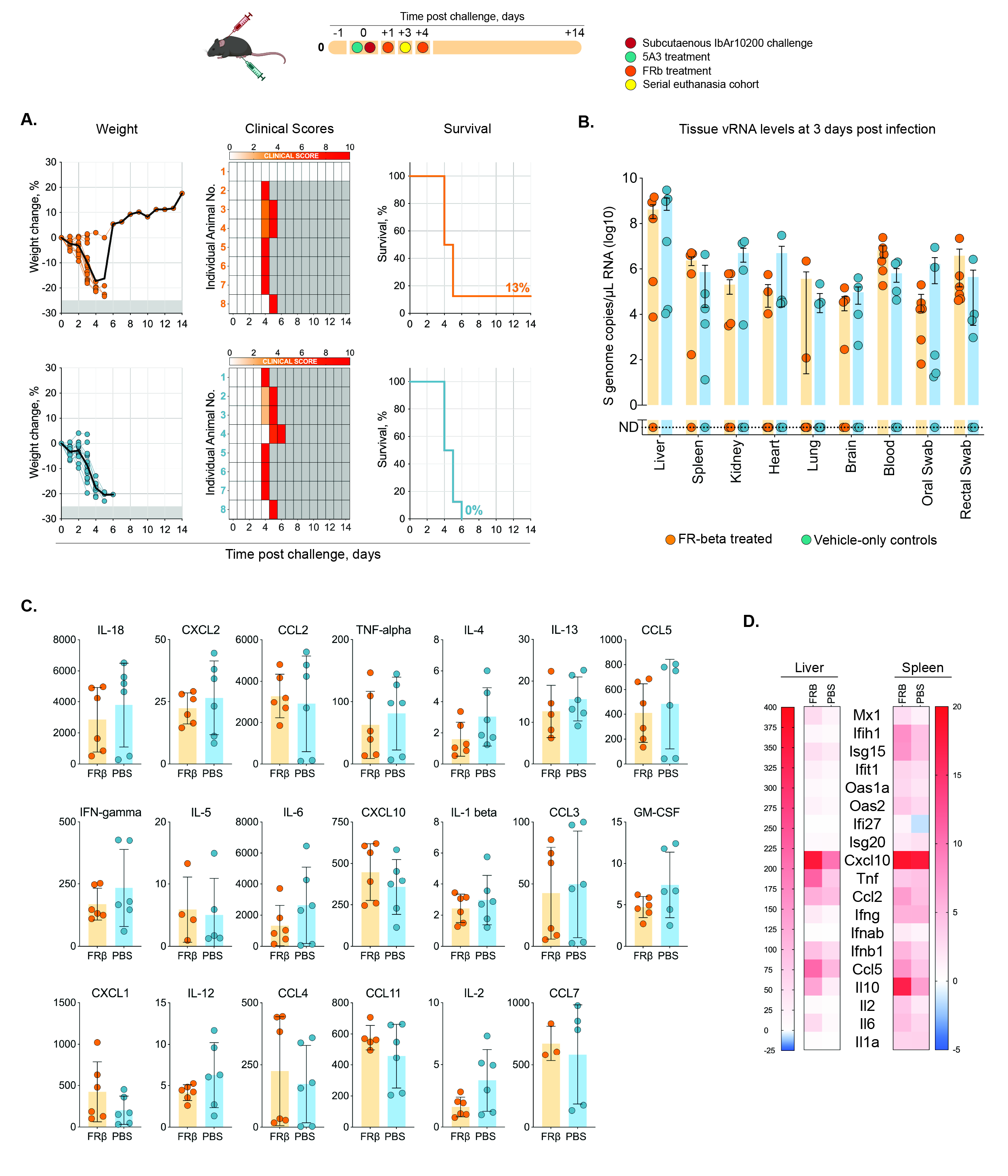
**

**Supplementary Figure 4. Anti-folate receptor beta monoclonal antibody does not reduce inflammation in CCHFV IbAr10200-infected mice.** Folate receptor beta (FR-beta) is highly and specifically upregulated on activated but not resting macrophages or other cell types [1,2] and has been the target of experimental treatments designed to reduce macrophage-associated inflammation underlying chronic diseases [1,3–7]. The anti-FR-beta antibody used here was shown to resolve rheumatoid arthritis symptoms in mice with no adverse off-target effects clinically or on other cell populations including dendritic, natural killer (NK), T, or B cells [5]. **(A)** C57BL/6J mice (12–16/group, mixed male and female, 6 weeks of age) were challenged SC with 100 TCID_50_ CCHFV IbAr10200 and simultaneously received 2.5 mg mAb 5A3. Cohorts of mice were treated IP with 400 µg/dose (approximately 20 mg/kg) of folate receptor beta (FR-beta) mAb or PBS alone (control cohorts) administered 1 and 4 days post infection (dpi). Treatment dosing and timing were determined from the effective and safe concentrations described for mice by Hu *et al.* (2019) [5]. Subsets of mice (6/group) were serially euthanized at 3 dpi to evaluate whether inflammation was reduced in FR-beta-treated versus control animals. Mice were monitored daily for weight loss and signs of clinical disease until the study endpoint of 3 or 14 dpi. All (8 of 8, 100%) PBS-treated control mice and 7 of 8 (87.5%) anti-FR-beta treated mice succumbed to infection. Time to death, weight loss, and presentation of clinical signs did not significantly differ between treated and control animals. We confirmed CCHFV infection in the single survivor from the anti-FR-beta-treated group via ELISA at 14 dpi (reciprocal endpoint dilution titer of 1:800 [IgM] and 1:51200 [IgG]). **(B)** Tissues including liver, spleen, ovary/testis (gonad), kidney, lung, heart, eye, brain, and whole blood, as well as oral and rectal swabs, were collected from mice euthanized at 3 dpi. Viral RNA was isolated and quantified via RT-qPCR using primers/probe specific for the open reading frame of NP of the CCHFV S gene segment; levels were not significantly different between treated and control cohorts. Individual animals are represented. Bars and error bars indicate the mean ± standard deviation (SD). Statistics were calculated using multiple t-tests (Mann Whitney); only significant results are reported. **(C)** Cytokine/chemokine responses in all mice euthanized 3 dpi were analyzed using the ProcartaPlex Mouse Th1/Th2 Cytokine and Chemokine 20-plex panel and 25 µL mouse plasma. Individual animals are represented. Bars and error bars indicate the mean ± standard deviation (SD). Statistics were calculated using multiple t-tests (Mann Whitney); only significant results are reported. **(D)** Gene expression profiles in the liver and spleen of all mice euthanized 3 dpi were analyzed using an RT^2^ Profiler PCR Array (Qiagen). Gene expression data are reported as fold change compared to tissue from age-matched uninfected C57BL/6J control animals. Plasma analytes and gene expression profiles in liver and spleen that were previously determined to be associated with fatality, were not significantly reduced in treated animals.

**References**

[1] Lu YJ, Wheeler LW, Chu H, et al. Targeting folate receptor beta on monocytes/macrophages renders rapid inflammation resolution independent of root causes. Cell Rep Med. 2021;2:100422.

[2] Chandrupatla DMSH, Molthoff CFM, Lammertsma AA, et al. The folate receptor β as a macrophage-mediated imaging and therapeutic target in rheumatoid arthritis. Drug Deliv Transl Res. 2019;9:366–378.

[3] Nagayoshi R, Nagai T, Matsushita K, et al. Effectiveness of anti–folate receptor β antibody conjugated with truncated Pseudomonas exotoxin in the targeting of rheumatoid arthritis synovial macrophages. Arthritis Rheum. 2005;52:2666–2675.

[4] Nagayoshi R, Nakamura M, Ijiri K, et al. LY309887, antifolate via the folate receptor suppresses murine type II collagen-induced arthritis. Clin Exp Rheumatol. 2003;21:719–725.

[5] Hu Y, Wang B, Shen J, et al. Depletion of activated macrophages with a folate receptor-beta-specific antibody improves symptoms in mouse models of rheumatoid arthritis. Arthritis Res Ther. 2019;21:143.

[6] Lu Y, Stinnette TW, Westrick E, et al. Treatment of experimental adjuvant arthritis with a novel folate receptor-targeted folic acid-aminopterin conjugate. Arthritis Res Ther. 2011;13:R56.

[7] Xia W, Hilgenbrink AR, Matteson EL, et al. A functional folate receptor is induced during macrophage activation and can be used to target drugs to activated macrophages. Blood. 2009;113:438–446.
